# Early Pulmonary Stress During Intra-Abdominal Hypertension and Hypervolemia: Surfactant Protein A1 and Multi-Compartment Biomarker Responses in Rats

**DOI:** 10.3390/life16071210

**Published:** 2026-07-22

**Authors:** Mert Canbaz, Halim Ulugöl, Gülsüm Karduz, Kübra Vardar, Mukadder Orhan Sungur, Evren Şentürk, Uğur Aksu, Pınar Fırat, Perihan Ergin Özcan, Fevzi Toraman, Mert Şentürk, on behalf of the Istanbul Research and Education in Anesthesia NETwork (IREANETwork) Experimental Working Group

**Affiliations:** 1 Department of Anesthesiology and Reanimation, Istanbul Faculty of Medicine, Istanbul University, Istanbul 34093, Turkey; mcanbaz@ku.edu.tr (M.C.); mukadder.orhan@gmail.com (M.O.S.); pergin@istanbul.edu.tr (P.E.Ö.); 2 Department of Anesthesiology and Reanimation, School of Medicine, Acibadem University, Istanbul 34752, Turkey; halimulugol@yahoo.com.tr (H.U.); ftoraman@gmail.com (F.T.); 3 Biology Department, Science Faculty, Istanbul University, Istanbul 34134, Turkey; gulsum.karduz@gmail.com (G.K.); vrdrkubra@gmail.com (K.V.); uguraksu@istanbul.edu.tr (U.A.); 4 Department of Anesthesiology and Intensive Care Medicine, School of Medicine, Koç University, Istanbul 34450, Turkey; esenturk@kuh.ku.edu.tr; 5 Department of Pathology, School of Medicine, Koç University, Istanbul 34450, Turkey; pfirat@ku.edu.tr

**Keywords:** intra-abdominal hypertension, hypervolemia, surfactant protein A1, bronchoalveolar lavage, permeability index, endothelial glycocalyx, oxidative stress, inflammatory biomarkers, pulmonary barrier

## Abstract

Conventional endpoints may overlook pulmonary biological stress during abdominal mechanical loading and volume expansion. This controlled experimental study evaluated whether intra-abdominal hypertension (IAH), hypervolemia (HV), or their combination induces early pulmonary biochemical, permeability, oxidative–ischemic, inflammatory, and structural responses, with particular focus on surfactant protein A1 (SP-A1). Thirty-five male Wistar rats were randomized to Sham, HV, IAH, or IAH + HV groups. IAH was induced by CO_2_ pneumoperitoneum at 12–15 mmHg, and HV by 6% hydroxyethyl starch infusion until hematocrit decreased to approximately 25%; animals were followed for 120 min. Physiological variables, blood gases, wet-to-dry ratio, ATS-based histopathology, and biomarkers in plasma, bronchoalveolar lavage (BAL) fluid, and lung tissue were assessed. The dominant finding was a compartment-specific SP-A1 response: plasma SP-A1 was highest in the IAH group, whereas BAL fluid SP-A1 increased in both HV and IAH groups. These changes occurred without significant alterations in MAP, HR, SpO_2_, wet-to-dry ratio, or ATS lung injury score. Permeability index was higher in HV-containing groups, and sialic acid and oxidative–ischemic markers showed compartment-dependent changes, whereas TNF-α, IL-6, HA, and ANP did not differ significantly. These exploratory, hypothesis-generating findings suggest that SP-A1 may reveal early pulmonary epithelial/barrier stress before overt physiological or structural injury becomes apparent.

## 1. Introduction

Intra-abdominal hypertension (IAH) and hypervolemia (HV) are clinically relevant stressors that may coexist during critical illness, major surgery, abdominal pathology, trauma, or aggressive fluid administration [1,2]. Although each condition can independently affect cardiopulmonary physiology, their coexistence may create a distinct biological setting in which abdominal mechanical compression and volume-related vascular stress interact. Increased intra-abdominal pressure (IAP) may impair venous return, alter organ perfusion, elevate the diaphragm, reduce thoracoabdominal compliance, and compromise respiratory mechanics [1,3]. Excessive intravascular volume expansion may further increase hydrostatic load, vascular distension, and endothelial barrier stress [4]. Because fluid resuscitation may worsen IAH, while IAH may increase the need for additional circulatory support, these processes may reinforce each other [1,2]. Previous experimental studies have shown that increased IAP, pneumoperitoneum, or sustained IAH can alter cardiopulmonary physiology, organ perfusion, pulmonary oxidative–inflammatory responses, and histological lung injury, whereas experimental positive or liberal-fluid models suggest that volume loading may impair respiratory or organ-level physiology without uniformly producing parallel edema or structural lung injury [3,5,6,7,8,9]. However, the early pulmonary consequences of combined IAH and HV remain insufficiently defined. In particular, it remains unclear whether the coexistence of abdominal mechanical loading and intravascular volume expansion produces early pulmonary biological signals before conventional physiological, oxygenation, edema, or histological endpoints become abnormal.

The lung is particularly vulnerable to this interaction because it is exposed to both abdominal-to-thoracic pressure transmission and volume-related vascular loading. IAH may promote atelectasis, reduce lung volume, impair respiratory compliance, and alter gas exchange, whereas positive fluid balance may worsen respiratory mechanics and oxygenation by reducing aerated lung volume and increasing pulmonary vascular and interstitial load [3,8,10]. Experimental data also suggest that IAH-related respiratory consequences may extend beyond lung mechanics, including diaphragm dysfunction and oxidative stress under severe acute pancreatitis-associated IAH conditions [11]. Importantly, early pulmonary biological stress may not be fully reflected by routine physiological or structural endpoints. Experimental studies suggest that cardiac output may be preserved despite ongoing organ injury, and that respiratory mechanics or oxygenation may change even when lung wet-to-dry ratio and histological injury scores remain comparable [5,8]. Thus, stable hemodynamic or oxygenation variables may not exclude early pulmonary biological stress.

Surfactant protein A (SP-A1) is a biologically relevant marker of alveolar epithelial and surfactant-system response. It is mainly produced by alveolar type II epithelial cells and contributes to surfactant homeostasis, innate immune regulation, and inflammatory modulation within the distal airspace. SP-A1 was selected as the primary biomarker of interest because it is closely linked to the alveolar epithelial–surfactant compartment, which may be affected early by changes in transpulmonary mechanics, alveolo-capillary permeability, and local airspace stress. Although epithelial injury biomarkers such as SP-D, RAGE, and CC16 have also been investigated in lung injury, SP-A1 was selected because the present study focused specifically on the alveolar epithelial–surfactant system and on compartment-specific BAL/plasma responses rather than on a broad comparison of epithelial injury biomarkers [12,13,14]. Altered SP-A1 expression or concentration has been associated with lung injury and may reflect changes in alveolar epithelial function or alveolo-capillary barrier integrity [12,15,16]. Recent evidence supports SP-A, together with SP-D, as a surfactant-related biomarker associated with alveolar epithelial injury and capillary–alveolar barrier disruption, while paired serum/BAL fluid measurements suggest that circulating and airspace surfactant proteins may provide distinct biological information [12,13,14]. Because SP-A1 can be measured in both bronchoalveolar lavage (BAL) fluid and plasma, compartment-specific assessment may help distinguish local alveolar/surfactant responses from broader epithelial–barrier involvement or systemic translocation [12,14]. This rationale is also supported by acute and perioperative lung-injury literature showing that SP-A changes may accompany histological injury, inflammatory cytokine responses, wet/dry lung injury indices, and laparoscopic surgery-related postoperative inflammatory lung injury markers [17,18]. Furthermore, circulating pneumoproteins are influenced not only by lung-derived flux but also by systemic clearance; CC16 appears more dependent on renal clearance, whereas circulating SP-A may more closely reflect lung-derived flux across the alveolo-capillary barrier [19]. Circulating surfactant proteins have also been evaluated in lung congestion and injury in acute heart failure, supporting their broader relevance to alveolo-capillary stress beyond classical inflammatory lung disease [20]. Thus, SP-A1 was not used as a stand-alone marker of established lung injury, but as a compartment-sensitive indicator of early epithelial/surfactant-system stress.

To characterize early pulmonary stress beyond SP-A1, we used an integrated pathway-based panel covering barrier permeability, glycocalyx/membrane disturbance, oxidative–ischemic stress, inflammation, volume-related neurohumoral activation, pulmonary edema, and structural injury. Barrier permeability was assessed using the BAL-to-plasma protein permeability index; glycocalyx/membrane disturbance using sialic acid and hyaluronic acid (HA); oxidative–ischemic stress using advanced oxidation protein products (AOPP), malondialdehyde (MDA), and ischemia-modified albumin (IMA); inflammation using tumor necrosis factor-α (TNF-α) and interleukin-6 (IL-6); and volume-related neurohumoral activation using atrial natriuretic peptide (ANP). Serum-free hemoglobin, wet-to-dry lung weight ratio, and histopathology were included to relate these early biochemical signals to hemolysis-associated vascular stress, pulmonary edema, and structural injury [4,6,21,22,23,24]. Therefore, by isolating IAH, HV, and their combined exposure within the same controlled animal model, this study aimed to determine whether these stressors induce early compartment-specific pulmonary responses, with a primary focus on SP-A1 in plasma and BAL fluid. We hypothesized that IAH and/or HV would induce early biochemical evidence of pulmonary stress, particularly involving SP-A1 and barrier-related markers, even before overt hemodynamic instability, sustained oxygenation impairment, pulmonary edema, or histologically evident lung damage. To our knowledge, previous experimental studies have not simultaneously evaluated SP-A1 in both BAL fluid and plasma under isolated and combined IAH and HV conditions. The novel contribution of this study is therefore the compartment-specific assessment of SP-A1 together with complementary barrier, oxidative–ischemic, inflammatory, and structural endpoints in a controlled model that separates IAH, HV, and their combined exposure.

## 2. Materials and Methods

### 2.1. Animals and Ethics

The study protocol was reviewed and approved by the Animal Experimental Ethics Committee of Acıbadem University Laboratory Animal Application and Research Center (approval number: HDK-2018/27). All experimental procedures were conducted in accordance with institutional and international guidelines for the care and use of laboratory animals. Thirty-five male Wistar Albino rats (350–450 g) were housed under standard laboratory conditions at 22 ± 2 °C on a 12 h light/dark cycle with free access to food and water. All animal experiments were designed, conducted and reported in accordance with the Animal Research: Reporting of In Vivo Experiments (ARRIVE 2.0) guidelines to ensure methodological transparency and reproducibility.

### 2.2. Anesthesia and Surgical Preparation

Anesthesia was induced with intraperitoneal xylazine (8 mg/kg) and ketamine (80 mg/kg). Rats were positioned supine on a heating pad to maintain body temperature at 37 °C. Pulse oximetry saturation probes (SpO_2_) were attached to the tail for continuous monitoring. Following tracheotomy, all animals were mechanically ventilated using a standardized volume-controlled ventilation protocol. Ventilator settings were kept identical across groups to minimize differential ventilatory exposure and were selected to maintain normocapnia during the experimental period: tidal volume 10 mL/kg, PEEP 5 cmH_2_O, respiratory rate 19 breaths/min, FiO_2_ 0.40, and inspiratory-to-expiratory ratio 1:2. Anesthesia was maintained with 1–2% isoflurane in an oxygen–air mixture using a calibrated vaporizer. The depth of anesthesia was verified intermittently using the tail pinch reflex. The right femoral artery was cannulated for continuous invasive blood pressure monitoring and arterial blood gas sampling, while the right femoral vein was catheterized for drug and fluid administration. Rocuronium (1 mg/kg, intravenous) was administered to maintain muscle relaxation. A 14-gauge intravenous cannula was inserted through the abdominal wall for CO_2_ insufflation and IAP monitoring. After a 20 min stabilization period, baseline blood samples were obtained for blood gas analyses.

### 2.3. Sample Size and Allocation Rationale

Group sizes were determined a priori during the ethical approval and experimental planning stage in accordance with ARRIVE 2.0 and the reduction principles of animal experimentation. Because reliable preliminary data for SP-A1 responses under isolated and combined IAH/HV conditions were not available, a formal SP-A1-based power calculation was not feasible. Therefore, the sample size calculation was based on an expected moderate-to-large difference in a representative biochemical endpoint from comparable experimental data. Assuming an approximately 16% between-group difference, α = 0.05, and β = 0.20, the required sample size was estimated to be approximately eight animals per active experimental group. The Sham group was kept smaller because it served as a baseline reference group without exposure to IAH or HV, whereas the main biological comparisons focused on the active intervention groups. A larger number of animals was allocated to the combined IAH + HV group because this condition included both interventions and represented the most procedurally complex experimental arm. This allocation was planned to preserve sufficient analyzable data for the combined-exposure condition while maintaining the overall number of animals as low as possible. Accordingly, comparisons involving the Sham group were interpreted cautiously.

Randomization was performed by a lot-drawing method using pre-prepared group labels in accordance with the planned unequal allocation ratio. Before the experiments, all group labels were pooled and thoroughly mixed, and each animal was sequentially assigned to Sham (n = 4), HV (n = 9), IAH (n = 9), or IAH + HV (n = 13) according to the drawn label. All animals underwent anesthesia, tracheotomy, vascular catheterization, and mechanical ventilation. After baseline measurements, group-specific interventions were initiated, and animals were followed for a total experimental period of 120 min.

**Sham:** No IAH or HV induction was performed, and animals were followed under the same experimental conditions.

**IAH:** Pneumoperitoneum was induced by CO_2_ insufflation to achieve an IAP of 12–15 mmHg, maintained with a laparoscopic insufflation device as previously described [6].

**HV:** HV was induced by intravenous infusion of 6% hydroxyethyl starch 130/0.4 through the femoral vein at a rate of 1 mL/min until the hematocrit decreased to approximately 25%, following established protocols [23]. Hematocrit level was monitored intermittently using arterial blood sampling and point of care analysis.

**IAH + HV:** Both interventions were applied after baseline measurements within the same experimental period. HV was induced using the same 6% hydroxyethyl starch 130/0.4 infusion protocol, and IAP was maintained at 12–15 mmHg throughout the 120 min experimental period.

### 2.4. Hemodynamic and Oxygenation Monitoring

Mean arterial pressure (MAP), heart rate (HR), and SpO_2_ were continuously monitored throughout the experimental period using tail-mounted pulse oximetry and invasive arterial pressure monitoring via the femoral artery. Data were recorded at baseline and at 15 min intervals until the end of the experiment.

### 2.5. Biochemical Analyses

Blood, BAL and lung tissue samples were collected at the end of the experimental period for biochemical evaluation. The samples were centrifuged and stored at −80 °C until analysis. Spectrophotometric assays were used to measure AOPP, MDA, sialic acid, IMA, and free hemoglobin, according to established protocols. AOPP levels were determined using a modified spectrophotometric method based on the Hanasand protocol. IMA was measured using a modified albumin cobalt-binding method based on the Bar-Or protocol. Sialic acid levels were determined spectrophotometrically according to the Sydow method. Serum free hemoglobin was quantified spectrophotometrically using an absorbance-based equation at 415, 380, and 450 nm, according to published protocols [25]. For parameters expressed relative to protein content, measured values were normalized to total protein concentrations for standardization purposes.

Enzyme-linked immunosorbent assay (ELISA) was performed to quantify ANP, SP-A1, TNF-α, IL-6, and HA concentrations in plasma, BAL fluid, and/or lung tissue homogenates using commercially available ELISA kits according to the manufacturer’s instructions (Elabscience Biotechnology Inc., Wuhan, China). In brief, samples and predetermined standards were introduced into microplate wells pre-coated with a capture antibody specific to each target analyte. This was followed by incubation with a biotinylated detection antibody and a streptavidin-conjugated enzyme. Following each incubation phase, wells were thoroughly washed to remove unbound components. Subsequently, a substrate solution was introduced, initiating a colorimetric reaction whose intensity was directly proportional to the analyte concentration. The enzymatic reaction was then terminated, and absorbance was measured with a microplate reader at the manufacturer-specified wavelength. Analyte concentrations were derived from a standard curve constructed using reference concentrations provided with each kit. Additionally, the permeability index was ascertained with the ratio of BAL fluid protein concentration to plasma protein concentration. Protein concentrations in samples were determined using a standard colorimetric Bradford protein assay, with bovine serum albumin (BSA) used as the standard.

### 2.6. Sample Collection and Tissue Processing

At the end of the two-hour experimental period, arterial blood was collected for blood gas and biochemical analyses. Animals were euthanized by exsanguination under deep anesthesia. The lungs and trachea were harvested for histopathological and biochemical studies. BAL fluid was obtained by instilling 5 mL of sterile saline through the trachea, which was subsequently aspirated and collected for biochemical evaluation.

### 2.7. Histopathological Evaluation

Lung tissues were fixed in 10% formalin, embedded in paraffin, sectioned, and stained with hematoxylin–eosin. Pulmonary edema was assessed by calculating the lung wet/dry weight ratio, obtained by dividing the wet weight measured immediately after harvesting by the dry weight after drying to a stable weight. All specimens were examined under light microscopy by a pathologist blinded to group allocation. Histopathological lung injury was evaluated according to the American Thoracic Society (ATS) workshop report on experimental acute lung injury in animals [26]. Twenty randomly selected high-power fields were assessed for each animal. Five histological components were scored: neutrophils in the alveolar space, neutrophils in the interstitial space, hyaline membranes, proteinaceous debris filling the airspaces, and alveolar septal thickening. Each component was scored per field using the ATS scoring criteria, and component scores were summed across the 20 evaluated fields for each animal. The overall ATS lung injury score was calculated using the following weighted formula: [(20 × neutrophils in the alveolar space) + (14 × neutrophils in the interstitial space) + (7 × hyaline membranes) + (7 × proteinaceous debris filling the airspaces) + (2 × alveolar septal thickening)]/(number of fields × 100). The resulting score ranges from 0 to 1, with higher values indicating more severe histological lung injury.

### 2.8. Statistical Analysis

All statistical analyses were performed using R version 4.4.1 (R Foundation for Statistical Computing, Vienna, Austria). The rstatix, car, and ggpubr packages were used for data processing and visualization. Normality of data distribution was assessed using the Shapiro–Wilk test and visual inspection of Q–Q plots. Homogeneity of variances was evaluated using Levene’s test. For normally distributed variables, one-way ANOVA followed by Tukey’s post hoc test was applied. For non-normally distributed variables, the Kruskal–Wallis test followed by Dunn’s post hoc test with Holm adjustment for multiple comparisons was used. For physiological and blood gas parameters measured at baseline and at the end of the experiment, group, time, and group × time interaction effects were evaluated using a repeated-measures framework. Between-group comparisons at baseline and at the final measurement were reported separately. Results are reported as mean ± standard deviation (SD) or median (IQR), depending on data distribution. To provide an estimate of precision, 95% confidence intervals were calculated using percentile bootstrap with 5000 resamples. Confidence intervals were calculated for the same group estimate used in the manuscript presentation: the mean for variables presented as mean values and the median for variables presented as median values. Because multiple biomarkers were analyzed, these analyses were considered exploratory and hypothesis-generating; pairwise comparisons were adjusted within each endpoint, but no additional global correction was applied across all biomarkers. All tests were two-tailed, and a *p* < 0.05 was considered statistically significant after adjustment where applicable.

## 3. Results

### 3.1. Systemic Hemodynamic, Oxygenation, and Arterial Blood Gas Parameters

Systemic hemodynamic, oxygenation, and arterial blood gas parameters at baseline and final measurement are presented in Table 1. MAP values did not differ significantly among groups at baseline or final measurement (*p* = 0.857 and *p* = 0.088, respectively), and no significant group × time interaction was observed (*p* = 0.648). HR values also showed no significant between-group differences at baseline or final measurement (*p* = 0.493 and *p* = 0.409, respectively), with no significant group × time interaction (*p* = 0.261). SpO_2_ values did not differ significantly among groups at baseline or final measurement (*p* = 0.967 and *p* = 0.189, respectively), and the group × time interaction was not significant (*p* = 0.145).

Among arterial blood gas parameters, pH, PCO_2_, HCO_3_^−^, and lactate values did not differ significantly among groups at either baseline or final measurement. Baseline PO_2_ values differed significantly among groups (*p* = 0.026), whereas final PO_2_ values did not (*p* = 0.302). A significant group × time interaction was observed for PO_2_ values (*p* = 0.013). Baseline BE values also differed significantly among groups (*p* = 0.010), while final BE values did not (*p* = 0.676), and the group × time interaction was not significant (*p* = 0.784).

### 3.2. ELISA-Measured Markers of Inflammation and Epithelial Injury

**Figure 1:**
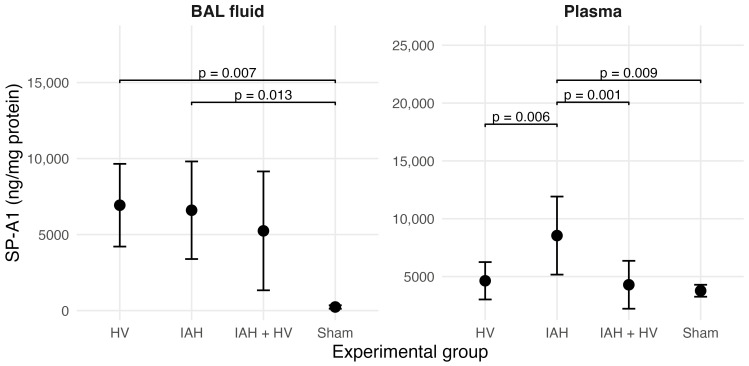
SP-A1 levels in BAL fluid and plasma (ng/mg protein). Dots and error bars represent mean ± SD. Brackets indicate statistically significant pairwise comparisons with adjusted *p* values. SP-A1, surfactant protein A1; BAL, bronchoalveolar lavage; HV, hypervolemia; IAH, intra-abdominal hypertension; SD, standard deviation.

Plasma and BAL fluid SP-A1 concentrations differed significantly among experimental groups (Figure 1). In plasma, SP-A1 concentrations were 4636.1 ± 1616.6 ng/mg protein in the HV group, 8544.0 ± 3373.9 ng/mg protein in the IAH group, 4291.0 ± 2070.4 ng/mg protein in the IAH + HV group, and 3774.8 ± 516.6 ng/mg protein in the Sham group (*p* < 0.001). Pairwise comparisons showed significantly higher plasma SP-A1 concentrations in the IAH group than in the HV, IAH + HV, and Sham groups (adjusted *p* = 0.006, *p* = 0.001, and *p* = 0.009, respectively). In BAL fluid, SP-A1 concentrations were 6930.8 ± 2720.3 ng/mg protein in the HV group, 6600.5 ± 3209.0 ng/mg protein in the IAH group, 5243.2 ± 3909.1 ng/mg protein in the IAH + HV group, and 236.2 ± 113.5 ng/mg protein in the Sham group (*p* = 0.009). Pairwise comparisons showed significantly higher BAL fluid SP-A1 concentrations in the HV and IAH groups than in the Sham group (adjusted *p* = 0.007 and *p* = 0.013, respectively).

No statistically significant between-group differences were observed in ELISA-based biomarker concentrations across the evaluated sample types (Table 2). ANP concentrations did not differ significantly among groups in plasma (*p* = 0.231) or heart tissue homogenate (*p* = 0.369). TNF-α concentrations did not differ significantly in plasma (*p* = 0.201), BAL fluid (*p* = 0.932), or lung tissue homogenate (*p* = 0.382). IL-6 concentrations were also similar among groups in plasma (*p* = 0.649), BAL fluid (*p* = 0.341), and lung tissue homogenate (*p* = 0.135). HA concentrations did not differ significantly among groups in plasma (*p* = 0.060), BAL fluid (*p* = 0.682), or lung tissue homogenate (*p* = 0.149).

### 3.3. Non-ELISA Biochemical Markers of Vascular Permeability and Oxidative Stress

**Figure 2:**
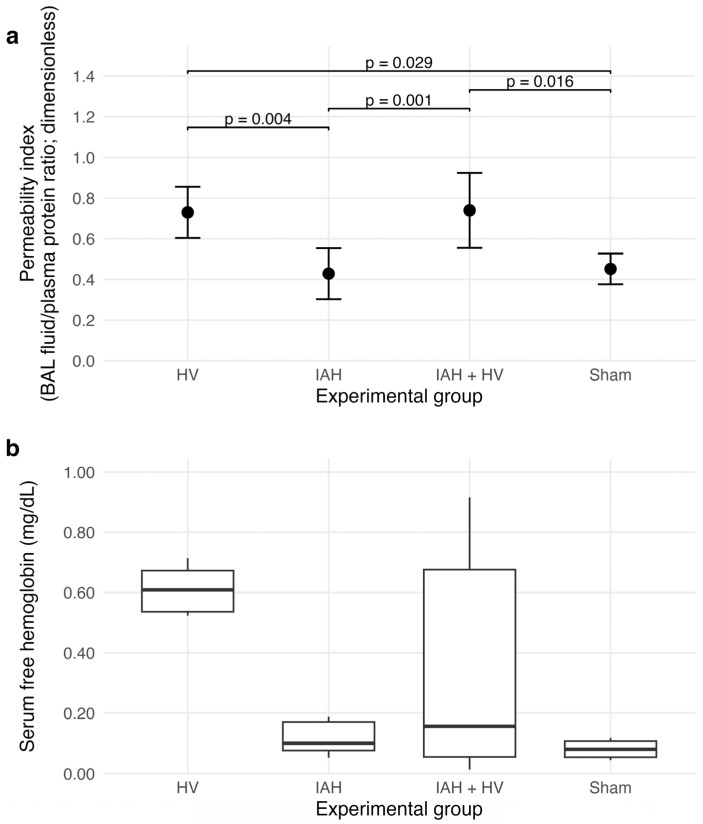
Vascular leakage and barrier integrity indicators. (**a**) Permeability index, calculated as the BAL fluid/plasma protein ratio; dots and error bars represent mean ± SD. (**b**) Serum free hemoglobin concentrations; boxes represent the median and IQR, and whiskers extend to 1.5 × IQR. Brackets indicate statistically significant pairwise comparisons with adjusted *p* values. BAL, bronchoalveolar lavage; HV, hypervolemia; IAH, intra-abdominal hypertension; IQR, interquartile range; SD, standard deviation.

Permeability index values differed significantly among experimental groups (*p* < 0.001; Figure 2a). The HV and IAH + HV groups showed higher mean permeability index values (0.730 ± 0.126 and 0.740 ± 0.185, respectively) than the IAH and Sham groups (0.429 ± 0.125 and 0.452 ± 0.076, respectively). Pairwise comparisons confirmed significantly higher permeability index values in the HV group compared with the IAH group (adjusted *p* = 0.004) and Sham group (adjusted *p* = 0.029), and in the IAH + HV group compared with the IAH group (adjusted *p* = 0.001) and Sham group (adjusted *p* = 0.016). No significant differences were observed between the HV and IAH + HV groups or between the IAH and Sham groups.

Serum free hemoglobin concentrations also differed among groups in the overall comparison (*p* = 0.045; Figure 2b). The highest median concentration was observed in the HV group (0.609 (0.536–0.673) mg/dL), followed by the IAH + HV group (0.156 (0.055–0.676) mg/dL), the IAH group (0.100 (0.076–0.171) mg/dL), and the Sham group (0.080 (0.054–0.107) mg/dL). However, pairwise comparisons did not show statistically significant differences after Holm adjustment; the lowest adjusted *p*-value was observed for the HV versus Sham comparison (adjusted *p* = 0.056).

**Figure 3:**
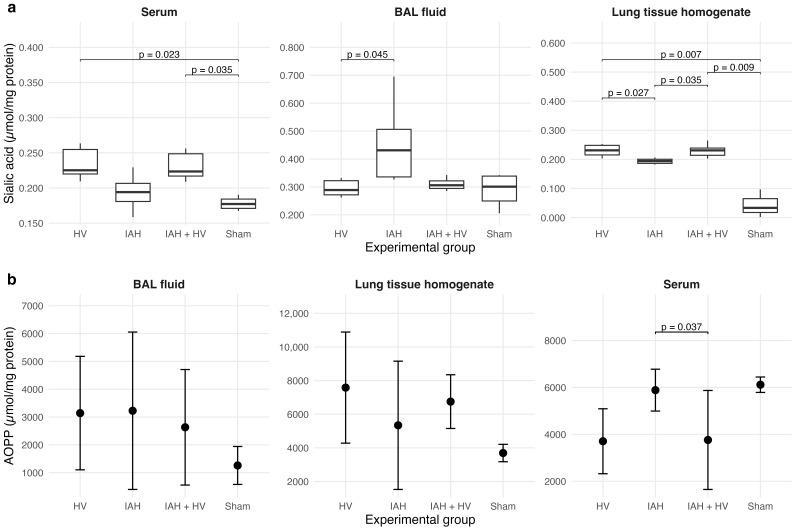
Markers of glycocalyx/membrane-associated injury and protein oxidation. (**a**) Sialic acid concentrations in plasma, BAL fluid, and lung tissue homogenate; boxes represent the median and IQR, and whiskers extend to 1.5 × IQR. (**b**) AOPP concentrations in BAL fluid, lung tissue homogenate, and plasma; dots and error bars represent mean ± SD. Brackets indicate statistically significant pairwise comparisons with adjusted *p* values. AOPP, advanced oxidation protein products; BAL, bronchoalveolar lavage; HV, hypervolemia; IAH, intra-abdominal hypertension; IQR, interquartile range; SD, standard deviation.

Sialic acid concentrations differed significantly among experimental groups in plasma, BAL fluid, and lung tissue homogenate (Figure 3a). In plasma, concentrations were 0.225 (0.220–0.255) µmol/mg protein in the HV group, 0.194 (0.181–0.207) µmol/mg protein in the IAH group, 0.223 (0.217–0.249) µmol/mg protein in the IAH + HV group, and 0.177 (0.171–0.184) µmol/mg protein in the Sham group (*p* = 0.007). Pairwise comparisons showed significantly higher plasma sialic acid concentrations in the HV and IAH + HV groups than in the Sham group (adjusted *p* = 0.023 and *p* = 0.035, respectively). In BAL fluid, sialic acid concentrations also differed among groups (*p* = 0.022), with concentrations of 0.289 (0.272–0.322), 0.431 (0.336–0.506), 0.306 (0.295–0.322), and 0.301 (0.250–0.339) µmol/mg protein in the HV, IAH, IAH + HV, and Sham groups, respectively. The only significant pairwise difference in BAL fluid was observed between the IAH and HV groups (adjusted *p* = 0.045). In lung tissue homogenate, concentrations were 0.231 (0.215–0.248), 0.194 (0.186–0.200), 0.230 (0.214–0.239), and 0.033 (0.018–0.065) µmol/mg protein in the HV, IAH, IAH + HV, and Sham groups, respectively (*p* < 0.001). Pairwise comparisons showed significant differences between HV and IAH (adjusted *p* = 0.027), HV and Sham (adjusted *p* = 0.007), IAH and IAH + HV (adjusted *p* = 0.035), and IAH + HV and Sham (adjusted *p* = 0.009).

AOPP concentrations differed significantly among groups in plasma (*p* = 0.008; Figure 3b). Plasma concentrations were 3706.23 ± 1385.08 µmol/mg protein in the HV group, 5885.39 ± 892.57 µmol/mg protein in the IAH group, 3760.95 ± 2110.17 µmol/mg protein in the IAH + HV group, and 6116.77 ± 328.90 µmol/mg protein in the Sham group. Tukey-adjusted pairwise comparisons showed a significant difference between the IAH and IAH + HV groups (adjusted *p* = 0.037), whereas no pairwise comparison involving the Sham group reached statistical significance after adjustment. AOPP concentrations did not differ significantly among groups in BAL fluid or lung tissue homogenate (*p* = 0.511 and *p* = 0.167, respectively).

**Figure 4:**
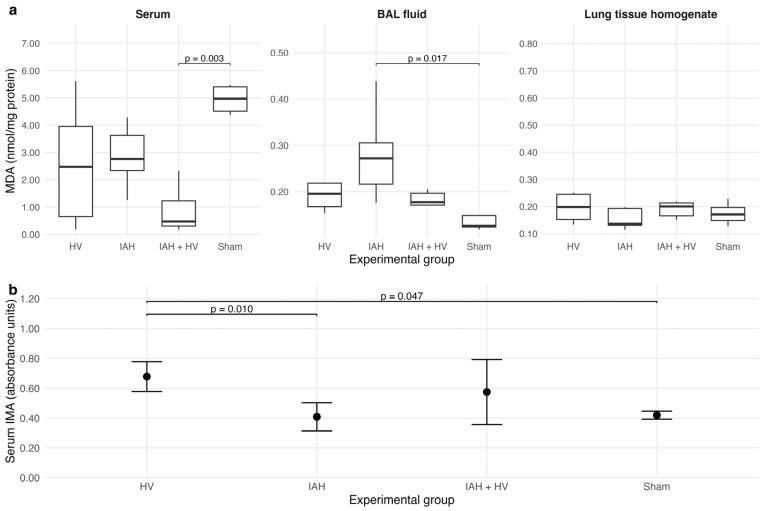
Oxidative stress and systemic ischemia markers. (**a**) MDA concentrations in plasma, BAL fluid, and lung tissue homogenate; boxes represent the median and IQR, and whiskers extend to 1.5 × IQR. (**b**) Plasma IMA levels; dots and error bars represent mean ± SD. Brackets indicate statistically significant pairwise comparisons with adjusted *p* values. MDA, malondialdehyde; IMA, ischemia-modified albumin; BAL, bronchoalveolar lavage; HV, hypervolemia; IAH, intra-abdominal hypertension; IQR, interquartile range; SD, standard deviation.

MDA concentrations differed significantly among experimental groups in plasma and BAL fluid, but not in lung tissue homogenate (Figure 4a). Plasma concentrations were 2.478 (0.655–3.953) nmol/mg protein in the HV group, 2.761 (2.339–3.630) nmol/mg protein in the IAH group, 0.472 (0.303–1.231) nmol/mg protein in the IAH + HV group, and 4.971 (4.512–5.408) nmol/mg protein in the Sham group (*p* = 0.006). Dunn–Holm pairwise comparisons showed a significant difference between the IAH + HV and Sham groups (adjusted *p* = 0.003). In BAL fluid, concentrations were 0.196 (0.168–0.219), 0.272 (0.217–0.305), 0.178 (0.171–0.196), and 0.126 (0.124–0.148) nmol/mg protein in the HV, IAH, IAH + HV, and Sham groups, respectively (*p* = 0.013), with a significant difference between the IAH and Sham groups (adjusted *p* = 0.017). Lung tissue homogenate MDA concentrations did not differ significantly among groups (*p* = 0.315).

Plasma IMA levels differed significantly among experimental groups (*p* = 0.008; Figure 4b). Plasma IMA levels were 0.678 ± 0.100 in the HV group, 0.408 ± 0.095 in the IAH group, 0.574 ± 0.211 in the IAH + HV group, and 0.419 ± 0.027 in the Sham group. Tukey-adjusted pairwise comparisons showed significantly higher plasma IMA levels in the HV group than in the IAH and Sham groups (adjusted *p* = 0.010 and *p* = 0.047, respectively).

To show the precision of the estimates given the small sample size, bootstrap 95% confidence intervals for biomarker and permeability endpoints are provided in Supplementary Table S1.

### 3.4. Effect of Intra-Abdominal Hypertension and Hypervolemia on Lung Histopathology

**Figure 5:**
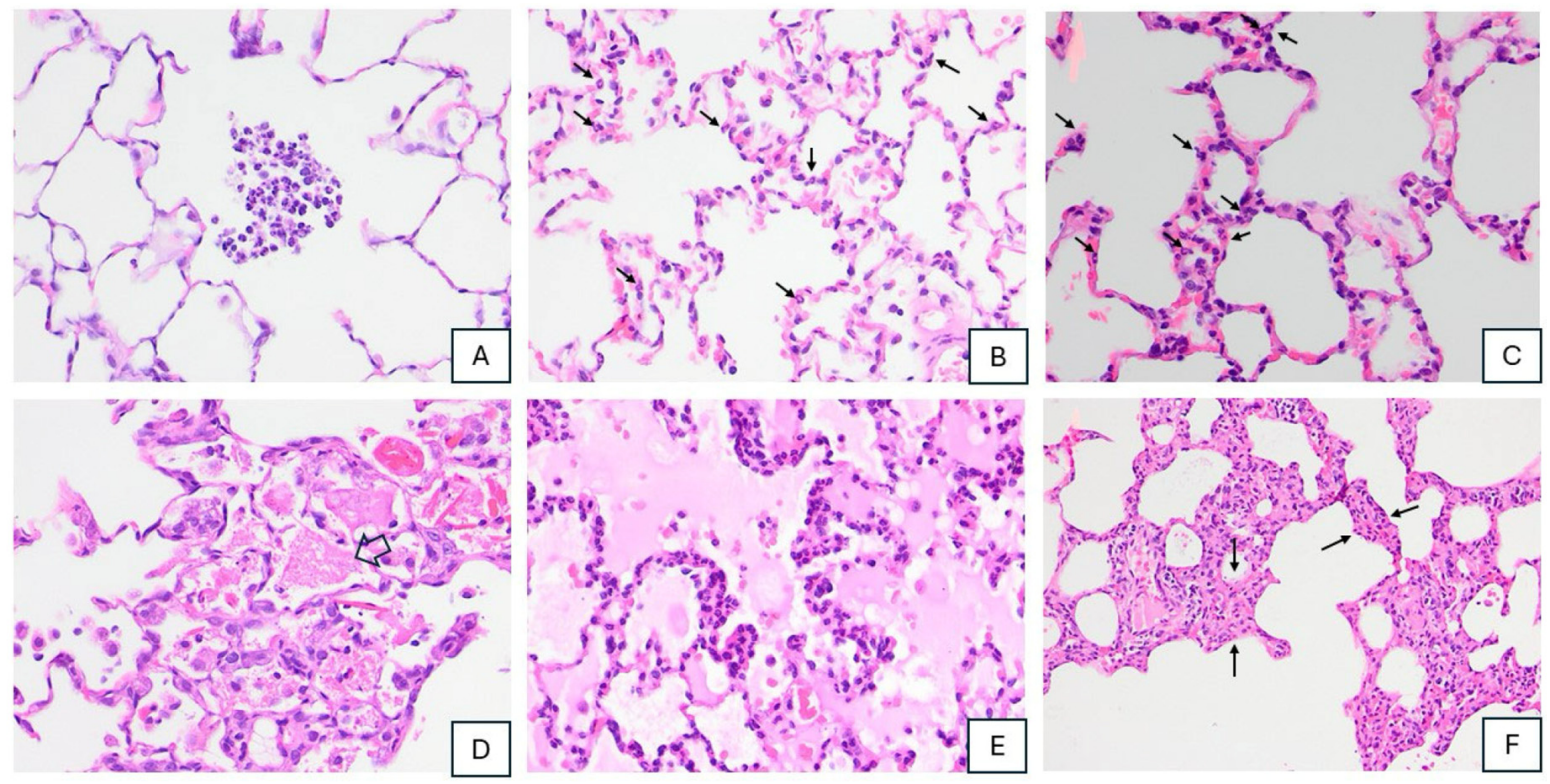
Representative histopathological lung sections illustrating ATS-based lung injury scoring features. (**A**). Neutrophils in alveoler spaces (<5) [H&E×400] (IAH group) (**B**). Neutrophils in the interstitium (<5) without alveolar septal thickening (<2×) [H&E×400] (IAH+HV group) (**C**). Neutrophils in the interstitium (>5) with 2×–4× alveolar septal thickening [H&E×400] (IAH group) (**D**). Proteinaceous debris filling the airspaces (>1) [H&E×400] (HV group) (**E**). Neutrophils in the interstitium (>5) with 2×–4× alveolar septal thickening + few neutrophils in the alveoler spaces (1–5) and some proteinaceous debris/edema fluid filling the alveoli (>1) [H&E×400] (IAH group) (**F**). Neutrophils in the interstitium (>5) and prominent alveolar septal thickening (>4×) [H&E×200] (IAH group). Black arrows in panels (**B**,**C**) indicate interstitial neutrophilic infiltration, whereas the black arrows in panel (**F**) indicate prominent alveolar septal thickening. The white outlined arrow in panel (**D**) indicates proteinaceous debris within the alveolar airspace.

Pulmonary edema and ATS-based histopathological assessment, including individual component scores and the overall ATS lung injury score, are summarized in Table 3. Neither the lung wet/dry weight ratio (HV: 6.91 ± 1.02, IAH: 7.43 ± 2.55, IAH + HV: 8.73 ± 1.44, Sham: 7.52 ± 1.01; *p* = 0.273) nor the overall ATS lung injury score (HV: 0.312 (0.278, 0.339), IAH: 0.268 (0.212, 0.323), IAH + HV: 0.202 (0.188, 0.237), Sham: 0.229 (0.207, 0.249); *p* = 0.057) showed a statistically significant between-group difference. Representative H&E-stained lung sections illustrating the main histopathological features used for ATS-based lung injury scoring are shown in Figure 5.

## 4. Discussion

The principal exploratory finding of this study was that short-term exposure to IAH and HV, either alone or in combination, elicited a selective pulmonary biochemical response without overt physiological or structural deterioration. Alterations in SP-A1, permeability index, sialic acid, and oxidative–ischemic stress-related markers indicate that epithelial and barrier-related perturbations may emerge before detectable changes in MAP, HR, SpO_2_, wet-to-dry ratio, or the overall ATS lung injury score. Rather than producing a uniform advanced lung injury phenotype within the 120 min experimental period, IAH and HV generated an early and compartment-specific pattern of pulmonary biological response. This dissociation highlights the potential value of biomarker-based assessment for detecting early lung stress that may not yet be apparent using conventional physiological or histological endpoints. However, because of the small sample size and unequal group sizes, these findings should be interpreted as hypothesis-generating rather than conclusive.

SP-A1 was the most consistent surfactant-related signal in this model and therefore represents the central biochemical finding of the study. Plasma SP-A1 was markedly higher in the IAH group than in the other groups, whereas BAL fluid SP-A1 was increased in both the HV and IAH groups compared with Sham. This compartment-dependent pattern is biologically plausible because SP-A-related proteins are closely linked to alveolar type II epithelial cell function, surfactant homeostasis, innate immune regulation, and alveolo-capillary barrier integrity [12,16,27]. Thus, the SP-A1 response appears to capture an early epithelial/surfactant-system signal that was not reflected by conventional physiological or histological endpoints. Importantly, these SP-A1 changes should not be interpreted as direct evidence of established functional lung damage, because oxygenation, wet-to-dry lung weight ratio, and the overall ATS lung injury score did not show corresponding overt impairment. Rather, the compartment-specific SP-A1 response may reflect an early biological change in the alveolar epithelial–surfactant system, including altered surfactant secretion/release and/or subtle epithelial–barrier stress before measurable physiological or structural injury becomes apparent. However, the present experimental design did not directly assess epithelial cell injury, tight-junction integrity, or ultrastructural alveolo-capillary barrier disruption. Therefore, SP-A1 changes should be interpreted as indirect, compartment-specific biochemical signals. These findings are consistent with early epithelial–surfactant-system stress, rather than direct evidence of epithelial injury or established barrier disruption. The divergence between plasma and BAL fluid findings may indicate that IAH primarily promoted systemic translocation or epithelial–barrier perturbation, a mechanism consistent with the concept that lung-derived proteins may appear in the circulation as markers of epithelial leakage, whereas both IAH and HV were sufficient to alter the local alveolar/surfactant compartment [19]. Previous studies also suggest that parallel assessment of serum/plasma and BAL fluid SP-A may provide different biological information, supporting the interpretation that circulating and airspace SP-A1 responses should not necessarily be expected to change in the same direction or magnitude [14]. Notably, the combined IAH + HV group did not show an additive increase in SP-A1, suggesting that the pulmonary response to simultaneous mechanical and volume-related stress may be nonlinear rather than simply cumulative. This pattern further supports the interpretation that SP-A1 responses should be considered within their compartmental context rather than as a uniform dose-dependent marker of injury severity.

The permeability index provided complementary evidence linking SP-A1 alterations to early barrier-related dysfunction. Whereas SP-A1 reflects a surfactant- and epithelial-associated response, the permeability index reflects BAL-to-plasma protein transfer and therefore provides an indirect estimate of alveolo-capillary protein leak. Interpreted together, these two findings suggest that early pulmonary stress in this model involved both epithelial/surfactant-system activation and barrier permeability changes. Permeability index was higher in the HV and IAH + HV groups, suggesting that volume-related stress may have contributed more prominently to alveolo-capillary protein leak. However, because HV was induced with 6% HES to a hematocrit of approximately 25%, hemodilution and changes in plasma protein concentration may have contributed to higher BAL/plasma protein ratios. Therefore, the permeability index should be interpreted cautiously in HV-containing groups. The absence of significant differences in wet-to-dry ratio and the overall ATS lung injury score indicates that this permeability signal did not progress to measurable pulmonary edema or overt structural damage within the experimental period. This pattern is consistent with the concept that functional or biochemical changes in barrier permeability may precede morphologically detectable lung injury. Moreover, experimental data on fluid balance and lung injury suggest that fluid-related physiological alterations may occur without parallel increases in wet-to-dry ratio or histological severity, supporting the interpretation that permeability-related markers and conventional structural endpoints may capture different stages of lung involvement [4,8]. Therefore, the combined interpretation of SP-A1 and permeability index strengthens the view that these markers may serve as early indicators of pulmonary epithelial-barrier stress before established edematous or histological lung injury develops.

The sialic acid findings support the an early surface-structure response, but they should not be interpreted as direct evidence of generalized glycocalyx injury. Plasma sialic acid was higher in the HV and IAH + HV groups than in Sham, and lung tissue concentrations were also increased in HV and IAH + HV. In BAL fluid, the highest values were observed in the IAH group, suggesting a compartment-specific response to abdominal mechanical loading and volume-related stress. Because sialic acid residues are terminal components of membrane-associated glycoconjugates and contribute to endothelial and epithelial surface charge, cell–cell interactions, and barrier stability, increased sialic acid may reflect early glycoconjugate perturbation or surface-layer remodeling [4,28]. In contrast, HA concentrations did not differ significantly among groups. Because HA did not change and other glycocalyx components were not directly assessed, the present findings should not be overinterpreted as broad glycocalyx disruption based solely on sialic acid. Instead, they suggest that sialic acid may capture a more selective or earlier surface-structure response, whereas detectable HA release may require more extensive shedding, stronger inflammatory activation, or a longer observation period [4,29].

Serum free hemoglobin should be interpreted cautiously as a supportive marker of hemolysis-related redox and endothelial stress rather than as a direct indicator of lung injury. Although free hemoglobin differed across groups at the global level, adjusted pairwise comparisons did not identify a robust group-specific separation; therefore, this finding should not be used to attribute the pulmonary biochemical response to a single experimental condition. Nevertheless, the numerically higher values observed in the HV group are biologically relevant, because hemodilution and volume expansion may disturb erythrocyte membrane integrity and increase circulating free hemoglobin [23]. Once released into the vascular compartment, free hemoglobin can scavenge nitric oxide, promote oxidative reactions through heme- and iron-dependent pathways, and contribute to endothelial activation and barrier dysfunction [30,31]. Accordingly, in the present study, serum free hemoglobin is best regarded as one component of the broader vascular and oxidative stress profile, but not as independent evidence of established pulmonary injury.

The oxidative–ischemic stress markers showed a heterogeneous and compartment-specific pattern rather than a uniform injury signature. MDA differed significantly in plasma and BAL fluid, but not in lung tissue homogenate, suggesting that lipid peroxidation-related responses varied according to the sampled compartment. The higher BAL fluid MDA observed in the IAH group is consistent with previous experimental data linking CO_2_ pneumoperitoneum and increased IAP to oxidative stress, reduced nitric oxide bioavailability, and inflammatory activation in lung tissue [6,7]. In contrast, the relatively high plasma MDA values in the Sham group should be interpreted cautiously. Because Sham animals still underwent anesthesia, tracheotomy, vascular catheterization, and mechanical ventilation, this group does not represent a completely naive control condition. Therefore, plasma MDA may partly reflect procedure-related systemic oxidative stress, while the lower values observed in some intervention groups may indicate compartmental redistribution, timing-related variability, or activation of endogenous antioxidant responses rather than absence of biological stress. A similar caution applies to plasma AOPP. Although plasma AOPP differed across groups, the only adjusted pairwise difference was observed between the IAH and IAH + HV groups, while comparisons involving the Sham group did not remain significant. Therefore, the relatively high plasma AOPP values should be interpreted as a nonspecific systemic protein oxidation signal rather than as a group-specific indicator of pulmonary injury. AOPP and IMA provide complementary information within this framework, reflecting oxidative protein modification and ischemia-related albumin alteration, respectively [32,33]. The higher serum IMA level in the HV group may indicate that volume-related vascular stress was accompanied by systemic ischemia–oxidation-related albumin modification. However, because IMA was measured only in plasma and was not paralleled by wet-to-dry or histopathological changes, this finding should be interpreted as a systemic oxidative–ischemic signal rather than direct evidence of pulmonary tissue injury. Taken together, these findings suggest that IAH and HV did not induce a homogeneous oxidative injury phenotype within 120 min, but generated a complex biochemical stress response that differed between the vascular, alveolar, and tissue compartments.

The absence of significant differences in TNF-α and IL-6 suggests that the biochemical changes observed in this model were not accompanied by a clearly detectable systemic or pulmonary cytokine response within the 120 min observation period. This finding does not exclude inflammatory involvement, but indicates that the experimental insult may have been too short or too moderate to generate a measurable cytokine-driven injury phenotype. Previous pneumoperitoneum models have shown that higher IAP levels or stronger inflammatory stimulation may increase TNF-α, IL-6, oxidative stress, and histological lung injury [6,7]. Therefore, the lack of cytokine elevation in the present study is consistent with an early-stage response in which epithelial, permeability-related, and oxidative–ischemic signals precede overt inflammatory amplification. Similarly, unchanged ANP and HA concentrations suggest that atrial stretch-mediated glycocalyx shedding was not the dominant pathway in this model. Although hypervolemia and fluid loading have been associated with ANP release and glycocalyx degradation in previous experimental and clinical settings, our findings indicate that pulmonary barrier perturbation may occur without a parallel increase in these later or more systemic markers [4,29].

The lack of significant differences in wet-to-dry ratio and the overall ATS lung injury score is central to the interpretation of this model. Although SP-A1, permeability index, sialic acid, and selected oxidative–ischemic stress markers suggested early pulmonary biological perturbation, these changes were not accompanied by measurable lung water accumulation or overall ATS-defined histological lung injury. However, the significant differences observed in selected individual histological components, particularly alveolar-space neutrophils and proteinaceous debris, suggest that focal or component-specific microscopic changes may have occurred despite the absence of a significant difference in the overall ATS lung injury score. This indicates that the biochemical either preceded, or remained below the threshold required for, established structural lung damage. Previous pneumoperitoneum studies show that injury intensity and exposure conditions determine whether biochemical alterations progress to histological injury, with higher IAP levels producing more pronounced oxidative, inflammatory, and histopathological changes, whereas lower-pressure exposure may produce limited or no structural injury [6,7]. In experimental lung injury models, surfactant-related and inflammatory biomarkers have also been shown to change in parallel with wet-to-dry ratio and pathological alterations, supporting their value as dynamic indicators of lung injury progression [17]. Similarly, experimental fluid-balance data suggest that physiological and biochemical changes may occur without proportional changes in wet-to-dry ratio or histological severity [8]. Therefore, the absence of edema or histological damage in the present study should not be interpreted as absence of pulmonary stress, but rather as evidence that the 120 min protocol captured an early phase of lung barrier perturbation before progression to overt tissue injury.

Physiological and blood gas findings should be interpreted in the context of the intensity and duration of the experimental exposure. MAP, HR, and SpO_2_ remained comparable among groups, indicating that IAH and/or HV did not produce overt systemic instability during the 120 min protocol. This is consistent with clinical data showing that elevated IAP can be tolerated under controlled procedural conditions without major hemodynamic or ventilatory instability [34]. Conversely, experimental models using higher IAP levels have demonstrated significant cardiopulmonary and metabolic effects, including changes in cardiac index, respiratory mechanics, PaO_2_, and bicarbonate, suggesting that the physiological consequences of IAH are pressure-, duration-, and model-dependent [3]. In addition, because IAH was induced using CO_2_ pneumoperitoneum, the observed responses in IAH-containing groups may reflect not only increased intra-abdominal pressure but also CO_2_-related effects on acid–base balance and pulmonary vascular tone. In our study, PaO_2_ differed among groups at baseline and showed a significant group × time interaction, but final PaO_2_ values were not significantly different. The baseline between-group difference in PaO_2_ appeared to reflect relatively lower pre-intervention values in the Sham group. Because this difference was observed before group-specific interventions and final PaO_2_ values were comparable among groups, it was interpreted as baseline variability rather than evidence of sustained oxygenation impairment or an intervention-related effect. Therefore, the PaO_2_ interaction should be interpreted cautiously and should not be taken as evidence of sustained between-group oxygenation impairment. Similarly, BE differed at baseline but not at the final measurement, and no group × time interaction was observed. Therefore, these findings should not be interpreted as sustained oxygenation failure or progressive metabolic deterioration. Rather, they indicate that the observed biochemical changes developed despite largely preserved systemic physiological and gas exchange variables. This further supports the concept that early pulmonary biological stress may be detectable before conventional monitoring parameters show clear deterioration.

## 5. Strengths and Limitations

This study has several strengths. First, it evaluated IAH, HV, and their combined exposure within the same controlled experimental model, allowing the pulmonary effects of mechanical abdominal loading and volume-related stress to be examined both separately and together. Second, the study integrated physiological variables, blood gas parameters, wet-to-dry ratio, histopathology, and a broad multi-compartment biomarker panel obtained from plasma, BAL fluid, and lung tissue homogenates. This design allowed early biochemical signals to be interpreted alongside conventional structural and physiological endpoints. Third, the short-term 120 min protocol was suitable for identifying early pulmonary biological responses before the development of advanced tissue injury.

Several limitations should also be acknowledged. The observation period was limited to 120 min; therefore, delayed cytokine activation, pulmonary edema, or histopathological injury may not have been captured. The Sham group underwent anesthesia, tracheotomy, vascular catheterization, and mechanical ventilation, but a completely naïve non-instrumented control group was not included; this may be relevant particularly for interpreting systemic oxidative stress markers such as plasma MDA. In addition, although several biochemical measurements were normalized to total protein concentrations, ELISA-based biomarker concentrations were not uniformly protein-normalized, and HES-related hemodilution and plasma protein changes may have influenced biomarker concentrations and the BAL/plasma protein-based permeability index. Moreover, hydroxyethyl starch may exert biological effects independent of volume expansion, including potential effects on endothelial barrier function, glycocalyx integrity, colloid oncotic pressure, inflammatory responses, and coagulation-related pathways. Therefore, the HV intervention should be interpreted as an HES-based hypervolemia model rather than as a pure volume-expansion model. Because IAH was induced by CO_2_ pneumoperitoneum, CO_2_-related effects on acid–base balance and pulmonary vascular tone may also have confounded the interpretation of IAH-specific responses. Direct measures of intravascular volume status or cardiac stretch, such as central venous pressure or echocardiographic indices, were not obtained, limiting mechanistic interpretation of the ANP findings. Finally, additional mechanistic markers such as nitric oxide pathway mediators, antioxidant enzyme activity, haptoglobin, hemopexin, syndecan-1, or direct glycocalyx imaging were not assessed. The analysis of multiple biomarkers increases the risk of type I error; therefore, isolated statistically significant findings should be interpreted cautiously and in the context of the exploratory study design. The small and unequal group sizes, particularly the limited number of animals in the Sham group, may reduce the precision of control comparisons and increase uncertainty around between-group estimates; therefore, the findings should be interpreted as exploratory and hypothesis-generating. Despite these limitations, the combined physiological, biochemical, and histological assessment supports the interpretation that IAH and HV may induce early pulmonary biological stress before overt structural injury becomes detectable.

## 6. Conclusions

This exploratory experimental study suggests that the pulmonary consequences of IAH and HV may begin as early biochemical biomarker alterations before becoming apparent as overt physiological or structural injury. Short-term exposure to IAH and HV, either alone or in combination, induced an early and compartment-specific pulmonary biochemical response without overt hemodynamic instability, sustained oxygenation impairment, pulmonary edema, or histologically evident lung injury. The observed changes in SP-A1, permeability index, sialic acid, and oxidative–ischemic stress-related markers suggest early biochemical signals involving the epithelial–surfactant system, barrier-related pathways, and surface-structure responses, rather than directly demonstrating established epithelial injury or structural barrier disruption. However, these findings should not be interpreted as definitive evidence of established early lung injury, but rather as early biological signals of pulmonary stress in this exploratory model. This interpretation should be considered particularly cautious because several conventional inflammatory, neurohumoral, edema-related, and structural markers, including TNF-α, IL-6, HA, ANP, wet-to-dry lung weight ratio, and histopathology, did not show corresponding overt changes. Therefore, SP-A1 should be regarded as a hypothesis-generating compartment-specific biomarker signal rather than definitive evidence of established functional or structural lung damage. These findings highlight the potential value of multi-compartment biomarker assessment for detecting early pulmonary stress during IAH and HV. Further studies with longer observation periods and additional mechanistic markers are needed to determine whether these early biochemical alterations progress to clinically relevant lung injury.

## Figures and Tables

**Table 1:** Systemic hemodynamic, oxygenation, and arterial blood gas parameters at baseline and final measurement.

Parameter	Group	Baseline Measurement	*p* Value	Final Measurement	*p* Value	Group × Time *p* Value
**MAP (mmHg)**	**Sham**	79.5 (70.2–89.0)	0.857	72.0 (51.0–76.5)	0.088	0.648
	**HV**	75.0 (65.5–94.0)		70.0 (58.5–81.5)		
	**IAH**	87.0 (69.0–106.0)		78.0 (74.0–83.5)		
	**IAH + HV**	78.0 (69.8–86.0)		94.0 (88.0–98.5)		
**HR (bpm)**	**Sham**	196.2 ± 58.1	0.493	234.0 ± 50.7	0.409	0.261
	**HV**	214.0 ± 47.2		179.4 ± 64.2		
	**IAH**	233.1 ± 34.4		223.0 ± 59.8		
	**IAH + HV**	202.5 ± 58.4		225.1 ± 59.0		
**SpO_2_ (%)**	**Sham**	98.0 (98.0–99.0)	0.967	97.0 (87.5–98.5)	0.189	0.145
	**HV**	95.0 (92.8–97.8)		100.0 (86.0–100.0)		
	**IAH**	96.0 (91.0–97.0)		92.5 (80.8–95.2)		
	**IAH + HV**	95.0 (92.0–98.2)		82.0 (72.0–90.8)		
**pH**	**Sham**	7.35 (7.33–7.36)	0.225	7.30 (7.20–7.35)	0.195	0.051
	**HV**	7.28 (7.27–7.42)		7.25 (7.20–7.33)		
	**IAH**	7.34 (7.24–7.42)		7.22 (7.12–7.27)		
	**IAH + HV**	7.43 (7.41–7.45)		7.10 (6.99–7.19)		
**PaCO_2_ (mmHg)**	**Sham**	58.6 ± 3.5	0.227	66.3 ± 40.0	0.717	0.442
	**HV**	53.8 ± 22.2		55.5 ± 35.1		
	**IAH**	45.6 ± 15.6		53.7 ± 18.4		
	**IAH + HV**	39.8 ± 10.9		67.1 ± 16.5		
**PaO_2_ (mmHg)**	**Sham**	201.0 (195.0–210.0)	**0.026**	464.0 (363.0–557.5)	0.302	**0.013**
	**HV**	550.0 (528.0–602.0)		326.0 (259.5–537.2)		
	**IAH**	475.0 (397.8–555.5)		507.5 (380.2–638.8)		
	**IAH + HV**	614.5 (449.0–647.0)		376.5 (307.0–532.2)		
**HCO_3_^−^ (mmol/L)**	**Sham**	27.0 (26.5–28.2)	0.304	24.9 (23.1–28.6)	0.591	0.924
	**HV**	27.2 (22.1–27.3)		22.1 (21.0–24.6)		
	**IAH**	24.9 (20.8–25.9)		20.5 (16.6–24.5)		
	**IAH + HV**	24.8 (23.7–26.5)		23.6 (17.4–24.5)		
**Lactate (mmol/L)**	**Sham**	0.6 (0.6–0.7)	0.077	1.3 (1.1–1.9)	0.191	0.193
	**HV**	0.9 (0.8–1.0)		1.3 (0.7–3.6)		
	**IAH**	1.1 (0.9–1.4)		1.6 (1.1–2.8)		
	**IAH + HV**	1.2 (1.0–1.5)		0.9 (0.8–1.1)		
**BE (mmol/L)**	**Sham**	2.2 ± 0.3	**0.010**	−2.3 ± 2.5	0.676	0.784
	**HV**	−0.9 ± 2.1		−6.0 ± 7.6		
	**IAH**	−1.8 ± 3.3		−7.8 ± 7.3		
	**IAH + HV**	1.0 ± 1.9		−7.7 ± 7.2		

Values are presented as mean ± SD or median (IQR), according to data distribution. Group sizes were Sham (n = 4), HV (n = 9), IAH (n = 9), and IAH + HV (n = 13). Baseline and final *p* values represent between-group comparisons at the corresponding time point; group × time *p* values represent interaction effects over time. Bold values indicate statistical significance (*p* < 0.05). BE, base excess; HCO_3_^−^, bicarbonate; HR, heart rate; HV, hypervolemia; IAH, intra-abdominal hypertension; IQR, interquartile range; MAP, mean arterial pressure; PaCO_2_, arterial partial pressure of carbon dioxide; PaO_2_, arterial partial pressure of oxygen; SD, standard deviation; SpO_2_, peripheral oxygen saturation.

**Table 2:** ELISA-based biomarker concentrations in plasma, BAL fluid, and lung tissue homogenates across experimental groups.

Biomarker	Sample Type	HV (n = 9)	IAH (n = 9)	IAH + HV (n = 13)	Sham (n = 4)	*p*-Value
**Atrial Natriuretic Peptide (ANP)**	**Plasma (pg/mL)**	19.1 ± 5.9	29.9 ± 14.5	23.4 ± 10.0	23.1 ± 6.7	0.231
	**Heart tissue (pg/mg protein)**	3.8 ± 1.5	4.3 ± 1.6	3.1 ± 2.1	4.6 ± 1.3	0.369
**Tumor Necrosis Factor-α (TNF-α)**	**Plasma (pg/mL)**	15.7 ± 2.0	18.7 ± 5.3	15.9 ± 4.0	23.4 ± 11.4	0.201
	**BAL fluid (pg/mL)**	33.1 ± 11.4	39.8 ± 21.1	37.5 ± 26.9	33.5 ± 22.7	0.932
	**Lung tissue (pg/mg protein)**	23.7 ± 11.4	22.2 ± 8.4	15.4 ± 2.9	19.1 ± 1.8	0.382
**Interleukin-6 (IL-6)**	**Plasma (pg/mL)**	2.3 ± 0.5	2.5 ± 1.0	2.0 ± 0.3	2.3 ± 0.6	0.649
	**BAL fluid (pg/mL)**	8.0 ± 5.0	16.9 ± 13.9	10.0 ± 4.2	11.1 ± 8.3	0.341
	**Lung tissue (pg/mg protein)**	7.1 ± 3.4	4.9 ± 1.8	3.9 ± 1.0	5.7 ± 2.2	0.135
**Hyaluronic Acid (HA)**	**Plasma (ng/mL)**	1.8 (1.3–1.8)	1.2 (1.1–1.4)	1.5 (1.5–1.7)	1.4 (1.2–1.6)	0.060
	**BAL fluid (ng/mL)**	5.8 (3.5–7.3)	3.7 (3.6–4.0)	4.0 (3.8–4.5)	4.8 (4.4–6.1)	0.682
	**Lung tissue (ng/mg protein)**	5.5 (4.6–5.7)	5.1 (4.7–5.3)	4.7 (4.5–4.9)	5.2 (5.0–5.9)	0.149

Values are presented as mean ± SD or median (IQR), according to data distribution. *p* values represent between-group comparisons. ANP, atrial natriuretic peptide; BAL, bronchoalveolar lavage; ELISA, enzyme-linked immunosorbent assay; HA, hyaluronic acid; HV, hypervolemia; IAH, intra-abdominal hypertension; IL-6, interleukin-6; IQR, interquartile range; SD, standard deviation; TNF-α, tumor necrosis factor-alpha.

**Table 3:** Pulmonary edema and ATS-based histopathological lung injury scores.

Parameter	HV (n = 9)	IAH (n = 9)	IAH + HV (n = 13)	Sham (n = 4)	*p*-Value
***Alveolar neutrophils***	10.0 (8.0, 13.5)	10.5 (8.2, 12.5)	7.0 (5.0, 10.0)	3.5 (1.8, 5.5)	**0.035**
***Interstitial neutrophils***	24.0 (21.0, 28.5)	23.0 (18.0, 25.2)	18.0 (15.0, 20.0)	23.0 (22.5, 23.8)	0.070
***Hyaline membranes***	0.0 (0.0, 0.0)	0.0 (0.0, 0.0)	0.0 (0.0, 0.0)	0.0 (0.0, 0.0)	-
***Proteinaceous debris***	5.0 (4.2, 5.8)	0.5 (0.0, 3.0)	0.0 (0.0, 0.0)	3.5 (2.8, 4.8)	**0.020**
***Alveolar septal thickening***	13.5 (9.8, 24.8)	4.5 (3.0, 17.8)	5.5 (4.0, 7.2)	13.0 (8.5, 16.5)	0.204
***ATS lung injury score***	0.312 (0.278, 0.339)	0.268 (0.212, 0.323)	0.202 (0.188, 0.237)	0.229 (0.188, 0.249)	0.057
***Lung wet/dry weight ratio***	6.9 ± 1.0	7.4 ± 2.6	8.7 ± 1.4	7.5 ± 1.0	0.273

Histopathological component scores and ATS lung injury score are presented as median (IQR), whereas lung wet/dry weight ratio is presented as mean ± SD. *p* values represent between-group comparisons. Bold values indicate statistical significance (*p* < 0.05). ATS, American Thoracic Society; HV, hypervolemia; IAH, intra-abdominal hypertension; IQR, interquartile range; SD, standard deviation.

## Data Availability

The data presented in this study are available from the corresponding author upon reasonable request. The data are not publicly available because they are derived from an experimental animal study and include institutional research records.

## Supplementary Materials

The following supporting information can be downloaded at: https://www.mdpi.com/article/10.3390/life16071210/s1, Supplementary Table S1: Bootstrap 95% confidence intervals for biomarker and permeability endpoints according to experimental group.

## Abbreviations

ANP, atrial natriuretic peptide; AOPP, advanced oxidation protein products; ARDS, acute respiratory distress syndrome; ARRIVE, Animal Research: Reporting of In Vivo Experiments; ATS, the American Thoracic Society; BAL, bronchoalveolar lavage; BE, base excess; CO_2_, carbon dioxide; ELISA, enzyme-linked immunosorbent assay; FiO_2_, fraction of inspired oxygen; HA, hyaluronic acid; HCO_3_^−^, bicarbonate; HES, hydroxyethyl starch; HR, heart rate; HV, hypervolemia; IAH, intra-abdominal hypertension; IAP, intra-abdominal pressure; IL-6, interleukin-6; IMA, ischemia-modified albumin; IQR, interquartile range; MAP, mean arterial pressure; MDA, malondialdehyde; PaCO_2_, arterial partial pressure of carbon dioxide; PaO_2_, arterial partial pressure of oxygen; PEEP, positive end-expiratory pressure; PMNL, polymorphonuclear leukocyte; SD, standard deviation; SP-A1, surfactant protein A1; SpO_2_, peripheral oxygen saturation; TNF-α, tumor necrosis factor-alpha; VV-ECMO, veno-venous extracorporeal membrane oxygenation.
