# Supplementary material for: Early Pulmonary Stress During Intra-Abdominal Hypertension and Hypervolemia: Surfactant Protein A1 and Multi-Compartment Biomarker Responses in Rats
Source: Life (Basel). 2026 Jul 22;16(7):1210. doi: 10.3390/life16071210 (PMC13412432; doi:10.3390/life16071210)
Supplement: Supplementary file 1 [file life-16-01210-s001.zip › life-4409793-supplementary.pdf]

Supplementary Table S1. Bootstrap 95% confidence intervals for biomarker and permeability endpoints according to experimental group

Outcome	Sample type / time point	Unit	CI type	HV	IAH	IAH + HV	Sham
SP-A1	BAL fluid	ng/mg protein	Bootstrap 95% CI for mean	5367.6–8689.3	4540.8–8685.5	2995.3–7865.0	158.2–343.4
SP-A1	Plasma	ng/mg protein	Bootstrap 95% CI for mean	3691.4–5678.7	6780.1–10927.2	3211.9–5467.4	3357.0–4174.0
ANP	Heart tissue	pg/mg protein	Bootstrap 95% CI for mean	2.9–4.9	3.3–5.3	2.0–4.3	3.9–5.7
ANP	Plasma	pg/mL	Bootstrap 95% CI for mean	16.6–29.6	22.3–39.7	18.2–28.7	16.4–29.7
TNF-α	BAL fluid	pg/mL	Bootstrap 95% CI for mean	27.4–46.0	29.3–83.8	18.0–54.9	19.6–59.7
TNF-α	Lung tissue	pg/mg protein	Bootstrap 95% CI for mean	18.7–37.1	17.1–26.5	14.1–17.2	17.9–25.6
TNF-α	Plasma	pg/mL	Bootstrap 95% CI for mean	13.2–16.7	15.5–22.7	14.5–33.4	11.6–34.3
IL-6	BAL fluid	pg/mL	Bootstrap 95% CI for mean	5.6–11.8	8.1–28.9	6.9–17.6	5.3–16.9
IL-6	Lung tissue	pg/mg protein	Bootstrap 95% CI for mean	5.5–13.2	4.0–5.8	3.6–5.1	4.5–11.0
IL-6	Plasma	pg/mL	Bootstrap 95% CI for mean	2.0–2.6	1.9–7.1	1.8–3.2	1.9–2.9
HA	BAL fluid	ng/mL	Bootstrap 95% CI for median	3.382–7.691	3.573–4.036	3.764–6.982	3.573–9.491
HA	Lung tissue	ng/mg protein	Bootstrap 95% CI for median	4.418–5.836	4.282–5.291	4.500–4.936	4.964–7.243
HA	Plasma	ng/mL	Bootstrap 95% CI for median	1.179–1.824	0.956–1.379	1.479–2.148	1.133–1.649
Sialic acid	Serum	µmol/mg protein	Bootstrap 95% CI for median	0.214–0.301	0.167–0.229	0.214–0.250	0.167–0.190
Sialic acid	BAL fluid	µmol/mg protein	Bootstrap 95% CI for median	0.265–0.369	0.336–0.572	0.285–0.343	0.206–0.342
Sialic acid	Lung tissue	µmol/mg protein	Bootstrap 95% CI for median	0.207–0.310	0.186–0.202	0.207–0.252	0.002–0.097
AOPP	BAL fluid	µmol/mg protein	Bootstrap 95% CI for mean	1756.3–4697.7	1637.2–4994.9	1658.1–4145.6	723.5–1805.2
AOPP	Lung tissue	µmol/mg protein	Bootstrap 95% CI for mean	5321.8–10098.1	3147.5–8053.6	5662.8–7752.0	3251.9–4131.4
AOPP	Serum	µmol/mg protein	Bootstrap 95% CI for mean	2678.1–4667.9	5249.5–6398.1	2429.7–5187.5	5807.7–6347.8
MDA	BAL fluid	nmol/mg protein	Bootstrap 95% CI for median	0.159–0.283	0.216–0.327	0.171–0.206	0.118–0.213
MDA	Lung tissue	nmol/mg protein	Bootstrap 95% CI for median	0.141–0.439	0.116–0.198	0.154–0.219	0.127–0.228
MDA	Serum	nmol/mg protein	Bootstrap 95% CI for median	0.248–4.895	2.118–3.843	0.260–2.333	4.375–5.478
IMA	Serum	absorbance units	Bootstrap 95% CI for mean	0.607–0.752	0.348–0.470	0.447–0.720	0.398–0.444
Serum free hemoglobin	Serum	mg/dL	Bootstrap 95% CI for median	0.377–0.699	0.076–0.188	0.017–0.698	0.044–0.118
Permeability index	BAL/plasma	dimensionless	Bootstrap 95% CI for mean	0.643–0.824	0.355–0.514	0.623–0.859	0.390–0.510

Values represent 95% confidence intervals only. Confidence intervals were calculated using percentile bootstrap with 5000 resamples. For variables presented as mean values in the manuscript, confidence intervals are provided for the mean. For variables presented as median values in the manuscript, confidence intervals are provided for the median. CI, confidence interval; HV, hypervolemia; IAH, intra-abdominal hypertension; BAL, bronchoalveolar lavage.
